# Comparison of the antimicrobial reduction effect of photodynamic inactivation with the addition of chlorophyll and curcumin photosensitizer in *Aggregatibacter actinomycetemcomitans* and *Enterococcus faecalis*

**DOI:** 10.12688/f1000research.128483.5

**Published:** 2025-11-14

**Authors:** Deny Arifianto, Suryani Dyah Astuti, Sarah Ratri Medyaz, Septia Budi Lestari, Samian Samian, Dezy Zahrotul Istiqomah Nurdin, Dita Ayu Hariyani, Yunus Susilo, Ardiansyah Syahrom

**Affiliations:** 1 Doctoral Degree, Faculty of Science and Technology, Airlangga University, Surabaya, East Java, 60115, Indonesia; 2 Department of Engineering, Faculty of Vocational, Universitas Airlangga, Surabaya, Indonesia; 3 Department of Physics, Faculty of Science and Technology, Airlangga University, Surabaya, East Java, 60115, Indonesia; 4 Magister of Biomedical Engineering, Faculty of Science and Technology, Airlangga University, Surabaya, East Java, 60115, Indonesia; 5 Faculty of Engineering, Dr Soetomo University, Surabaya, East Java, 60115, Indonesia; 6 Medical Devices and Technology Centre, Universiti Teknologi Malaysia, Johor Bahru, Johor, 81310, Malaysia

**Keywords:** Aggregatibacter actinomycetemcomitans, Enterococcus faecalis, photodynamic inactivation, diode laser, diseases, periodontitis, curcumin, chlorophyll

## Abstract

**Background:**

Antibiotic resistance in oral pathogens such as *Aggregatibacter actinomycetemcomitans* and *Enterococcus faecalis* necessitates alternative antimicrobial strategies. Photodynamic inactivation (PDI) is a promising approach that utilizes a photosensitizer (PS), light, and oxygen to generate cytotoxic reactive oxygen species. This study evaluated the efficacy of two natural photosensitizers, curcumin and chlorophyll (from Medicago sativa L.), for the photodynamic inactivation of these bacteria.

**Methods:**

Planktonic cultures of *A. actinomycetemcomitans* and *E. faecalis* were subjected to PDI using a 405 nm diode laser at various energy densities. The bacteria were treated with either curcumin (1.6 mg/mL) or chlorophyll (1.6 mg/mL) prior to laser irradiation. Bacterial viability was assessed using the colony-forming unit (CFU) assay. Groups without PS and without light served as controls.

**Results:**

At an energy density of 1.59 J/cm ^2^, laser irradiation resulted in a reduction of 35.8% and 36.7% for *A. actinomycetemcomitans* and *E. faecalis*, respectively. The addition of chlorophyll significantly enhanced bacterial reduction to 64.4% and 69.3%. Curcumin demonstrated superior efficacy, achieving bacterial reduction of 89.8% and 89.4% for *A. actinomycetemcomitans* and *E. faecalis*, respectively. The differences between the PS groups and the control groups were statistically significant (p < 0.05).

**Conclusions:**

Curcumin is a significantly more effective natural photosensitizer than chlorophyll from Medicago sativa L. for the photodynamic inactivation of *A. actinomycetemcomitans* and *E. faecalis*. These findings point to the prospect of curcumin-based PDI as a potent therapeutic alternative for combating resistant oral infections.

## Introduction

The oral cavity is one of the most important parts of the body that must be maintained. Infectious diseases of the teeth and mouth that are often found are periodontitis and endodontics. Periodontitis is a bacterial infection of the teeth that causes inflammation of the supporting tissues of the teeth, which include the gingiva, ligaments, cement, and alveolar bone. ^1^ Periodontitis is caused by pathogenic bacteria, predominantly gram-negative, anaerobic, or microaerophilic in the subgingival area. ^2^ *Aggregatibacter actinomycetemcomitans* bacteria are found in dental plaque, periodontal pockets, and buccal mucosa in up to 36% of the normal population. ^3^ *Aggregatibacter actinomycetemcomitans* bacteria can infect patients when the human immune system decreases and inhibits other organisms’ growth in the oral mucosa, teeth, and nasopharynx.

In general, gram-positive bacteria, *Enterococcus faecalis* ( *E. faecalis*), are found in the root canals of teeth. The bacterium *Enterococcus faecalis* is ovoid, with a diameter between 0.5 and 1 μm. ^4^ These bacteria are facultative anaerobes and can survive in extreme environments such as highly alkaline pH and high salt concentration conditions. The number of these bacteria in the human body can be minimized by paying attention to the food consumed and environmental conditions such as humidity. Furthermore, *E. faecalis* bacteria resist calcium hydroxide and antibiotics such as tetracycline. ^5^ Systemic treatment in the form of antibiotics has been widely used to treat periodontitis. However, several studies have reported cases of antimicrobial resistance to certain types of antibiotics. ^6^ So alternative therapy is needed that is effective and does not cause antibiotic resistance. ^7^ Therefore, the recommended alternative therapy in this study is photodynamic inactivation (PDI).

Periodontitis, a chronic inflammatory disease triggered by dysbiotic oral microbiome, has implications that extend far beyond the oral cavity. A recent bibliometric analysis highlights the deep and growing research interest in the strong relationship between periodontitis and systemic diseases, such as diabetes, cardiovascular disease, and adverse pregnancy outcomes. ^8^ This well-established systemic relationship confirms that effective periodontitis management is not merely a dental concern but a critical component of overall public health. Consequently, there is an urgent need to develop innovative and effective therapeutic strategies to control periodontal biofilm and reduce the associated local and systemic inflammatory burden. Conventional treatments for inflammatory conditions, including peri-implant mucositis, often consist of non-surgical mechanical debridement enhanced with antiseptics like chlorhexidine. ^9^ The limitations of these conventional treatments emphasize the necessity for exploring alternate therapy methods, including antimicrobial photodynamic inactivation.

Photodynamic inactivation (PDI) is a method of inactivating microorganisms by utilizing light to activate a photosensitizer (PS) agent that produces reactive oxygen species (ROS), causing cell lysis. ^10^^,^^11^ The suitability of the light spectrum with the PS absorption spectrum is the key to photophysical reactions, namely the absorption of light energy by PS agents, which will trigger photochemical and photobiological reactions to produce antimicrobial effects ^12^^,^^13^ and biomodulation. ^14^ PS is a light-sensitive molecule that plays a role in absorbing light energy. ^15^ PS is divided into two types, namely endogenous and exogenous photosensitizers. The addition of exogenous PS aims to increase the effectiveness of light energy absorption. ^16^ Some natural ingredients that are exogenous PS include chlorophyll and curcumin. Chlorophyll is a green substance found in green plants that photosynthesizes. ^17^ In photosynthesis, chlorophyll acts as a light catcher, energy transfer, and light conversion and can absorb a maximum wavelength range of 400-700 nm. ^18^ Chlorophyll acts as a photosensitizer because it is naturally able to absorb light. ^19^ The chemical structure of chlorophyll consists of a porphyrin ring that serves as the core framework and long hydrophobic side chains. The bioactivity of chlorophyll is due to its ability to act as an antioxidant, antimutagen, and anticarcinogen. Its unique chemical structure allows chlorophyll to capture harmful free radicals, reduce DNA damage, and modulate cellular processes involved in disease progression. In addition, its hydrophobic side chains facilitate interactions with biological membranes, affecting cellular uptake and signaling pathways. ^20^

Curcumin is a curcuminoid compound with yellow pigment in turmeric rhizome, which is antitumor, antioxidant, anticarcinogenic, anti-inflammatory, antiviral, antifungal, antispasmodic, and hepatoprotective. ^21^ The absorption spectrum of curcumin is in the wavelength range of 375-475 nm. ^22^ The advantage of curcumin as PS is that there are reactive functional groups, including ketone and phenol groups, that play a role in phototherapeutic activity. Phenol groups play an important role in the interaction of curcumin and the biological membranes of bacteria because the attachment of hydrogen groups plays an important role. ^23^

Previous studies have reported the effectiveness of using PS chlorophyll in alfalfa leaves with a blue LED activator of 20.48 J/cm ^2^ for the inactivation of *A. actinomycetemcomitans* bacteria by 81%. ^24^ The results of another study with the addition of PS curcumin and diode laser activator 403 nm 15.83 J/cm ^2^ in *Staphylococcus aureus* resulted in a mortality rate of 85.48%. ^23^ Then, another study using curcumin and blue LEDs on *S. aureus* bacteria resulted in a mortality rate of 91.49%. ^25^ Continuing previous studies, ^21^^,^^22^^,^^24^ this study aims to compare the effectiveness of antimicrobial reduction from photodynamic inactivation (PDI) on bacteria *A. actinomycetemcomitans* and *E. faecalis* with PS curcumin and chlorophyll Medicago sativa L. using a 405 nm diode laser. Diode laser irradiation was carried out at various lengths of irradiation time, namely 30, 60, 90, 120, 150, and 180 seconds. In addition, this study aims to determine the effective laser irradiation time to reduce the bacteria *A. actinomycetemcomitans* and *E. faecalis* with PS curcumin and chlorophyll Medicago sativa L.

## Methods

### Bacterial culture

Bacteria *A. actinomycetemcomitans* ATCC 43718 and *E. faecalis* ATCC 29212 were cultured in tryptone soy broth (TSB). Then, it was incubated for 24 hours at 37°C until the colonies reached ~10 ^8^ CFU/mL or 1.0 McFarland standard.

### Photosensitizer (PS)

Chlorophyll was extracted from Medicago sativa L (K-Link liquid, Indonesia) and Curcuma standard (Sigma Aldrich) with a concentration of 1.6 mg/mL diluted with sterile normal saline. The absorption spectra of chlorophyll and curcuma were measured using a Shimadzu UV-VIS 1800 spectrophotometer.

### Light source

The light source of a diode laser is 405 nm, and characterization was carried out using Jasco CT-10 monochromators to determine the peak wavelength. The power output was 2.49 mW, measured with the power meter OMM-6810B-220V. The spot beam area size is 0.28 cm ^2^. Diode laser irradiation was carried out with variations in the length of the irradiation time of 30, 60, 90, 120, 150, and 180 seconds. The energy density value can be calculated using equation 1 ^12^:

$$\text{Energy Density}\;\left(J.{\mathrm{cm}}^{-2}\right)=\text{Intensity}\;\left(W.m^{-2}\right)\times \text{Irradiation Time}\;\left(s\right)$$ (1)

### PDI treatment

The treatment samples consisted of two types of bacteria, *A. actinomycetemcomitans* and *E. faecalis.* The bacterial PDI treatment consisted of a negative control group without treatment (T0), a positive control group with the addition of chlorophyll and curcumin (T1), a 405 nm diode laser treatment group at various energy densities of 0.26; 0.53; 0.79; 1.06; 1.32; 1.59 J/cm ^2^ (S1), a 405 nm diode laser treatment group with the addition of chlorophyll Medicago sativa L 1.6 mg/ml (S2), and a 405 nm diode laser treatment group with the addition of curcumin PS 1.6 mg/ml (S3). In groups S2 and S3, samples were given chlorophyll or curcumin, and they were incubated for 10 minutes, then irradiated with a 405 nm diode laser with an exposure time of 30, 60, 90, 120, 150, and 180 seconds. The treated samples were grown on TSA media, incubated for 24 hours at 37°C, and the number of bacterial colonies grown was counted by the total plate count (TPC) method.

### Statistical analysis

CFU/mL was calculated for each treatment using equation 2. Furthermore, the percentage of bacterial reduction was calculated using equation 3 based on the control group. The results of bacterial reduction were statistically analyzed by a two-way Anova factorial test using IBM SPSS Statistics Version 21 to determine the effect of each factor and its interaction between factors. The test usually requires distributed and at least interval scale data, with significant differences determined as P<α=0.05. Tukey’s post hoc test and Kolmogorov-Smirnov test were used to check the normality of the data.

$$\frac{\mathrm{CFU}}{\mathrm{ml}}=\frac{\text{number of colonies}\times \text{dilution factor}}{\text{volume of culture plate}}$$ (2)

$$\text{\%Viability reduction of bacteria}=\left[\frac{\sum \frac{\mathrm{CFU}}{\mathrm{ml}}\;\left(\text{control}\right)-\sum \frac{\text{kCFU}}{\mathrm{ml}}\;\left(\text{treatment}\right)}{\sum \frac{\mathrm{CFU}}{\mathrm{ml}}\;\left(\text{control}\right)}\right]\times 100\%$$ (3)

## Results

**Figure 1.**
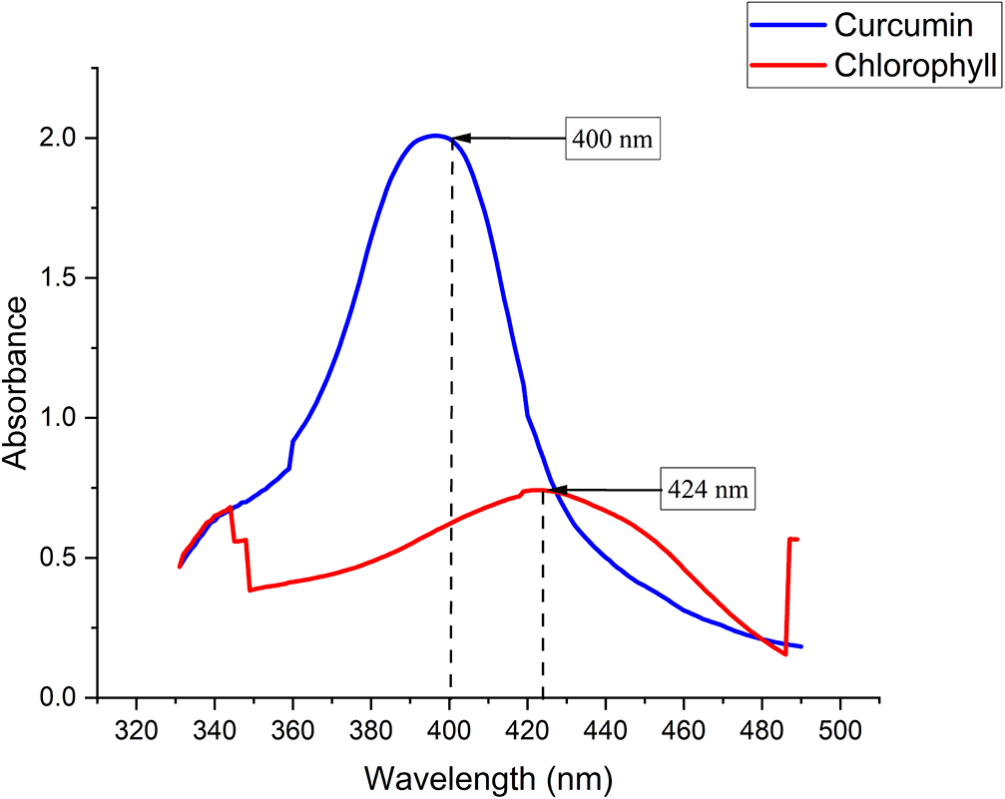
Graph of curcumin and chlorophyll absorption spectrum.

The chlorophyll and curcumin extracts were tested using UV-Vis at a wavelength range of 325 nm to 705 nm to determine the absorption spectrum of light. Then, the results of characterizing the absorption spectrum of chlorophyll and curcumin to light are obtained, as shown in Figure 1. Based on Table 1, the characterization results show the peak wavelength of the diode laser at 405 nm with the stability of the output power at a distance of 1 cm (2.49 ±0.07) mW. The temperature characterization showed the optimum temperature stability (26.60±0.01) °C for bacterial growth. Thus, the irradiation energy density of the diode laser is 405 nm with an output power of 2.49 mW and a beam area of 0.28 at various exposure times (30, 60, 90, 120, 150, 180) seconds are 0.26, 0.53, 0.79, 1.06, 1.32, and 1.59 J/cm ^2^.

**Table 1.** Laser energy density at various time exposure.

Laser parameters	
Parameter	Value
Emitter type	Laser Diode
Center wavelength	405 ± 0,02 nm
Operating mode	Continuous wave (CW)
Polarization	Linear
Beam spot size at target	≈ 2.80 ± 0.01 mm ^2^
Beam divergence	≈ 12 ^o^ parallel to beam ≈ 26 ^o^ perpendicular to the beam
Application technique	Distance 1 cm
Aperture diameter	1.89 mm
Power	2.49 ± 0,01 mW
Beam shape	elliptical
Variation in laser exposure time	30; 60; 90; 120; 150 and 180 s
Spectral bandwidth	10 nm
Average radiant power	2.49 ± 0,01 mW
Variation in radiant exposure/energy density	0.26; 0.53; 0.79; 1.06; 1.32; 1.59 J/cm ^2^
Area irradiated	2.80 mm ^2^
Variation in radiant energy	0.075; 0.15; 0.22; 0.29; 0.37; 0.45 J

**Figure 2.**
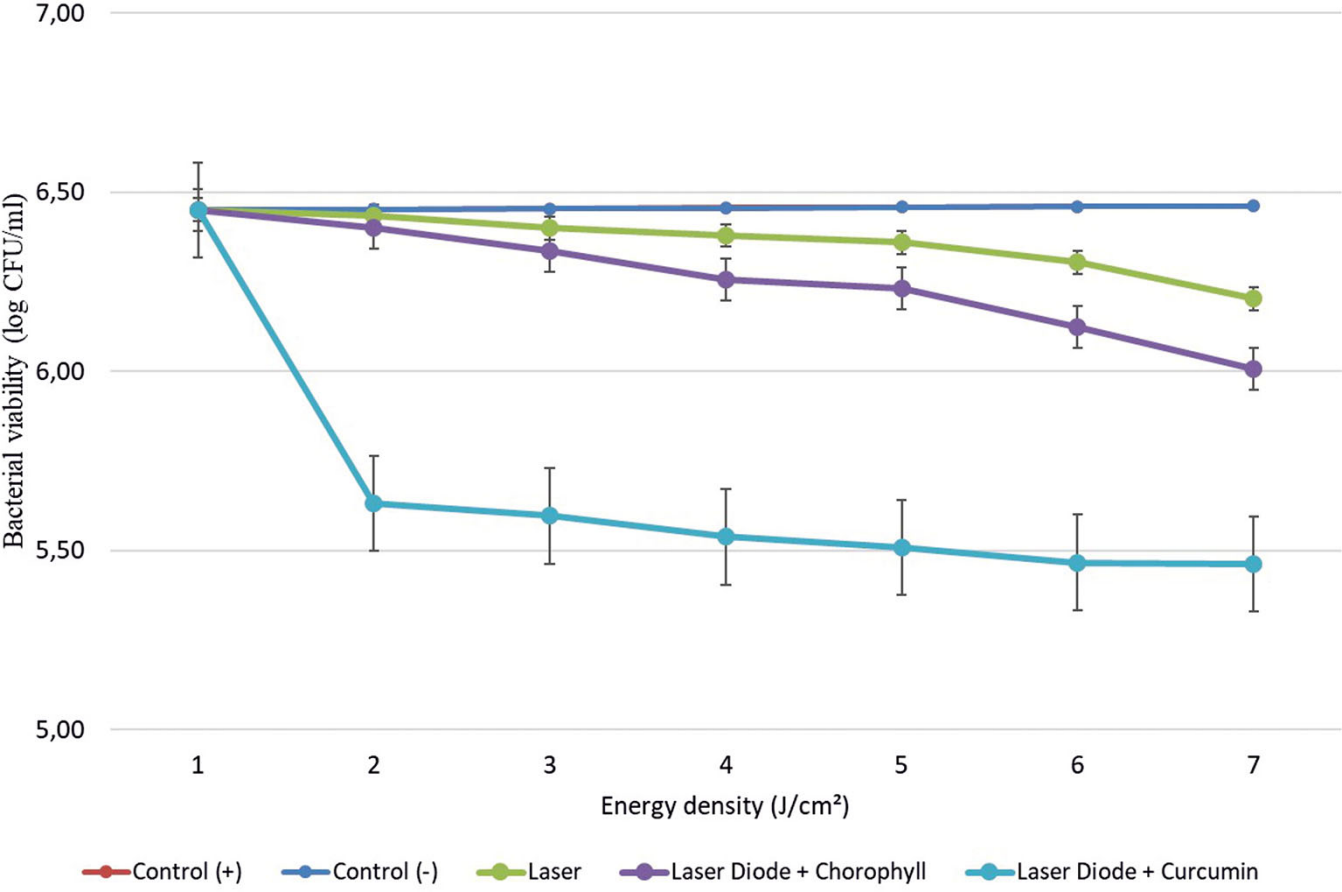
Graph of bacterial viability of *A. actinomycetemcomitans* in various treatments with and without the addition of curcumin and chlorophyll Medicago sativa L.

**Figure 3.**
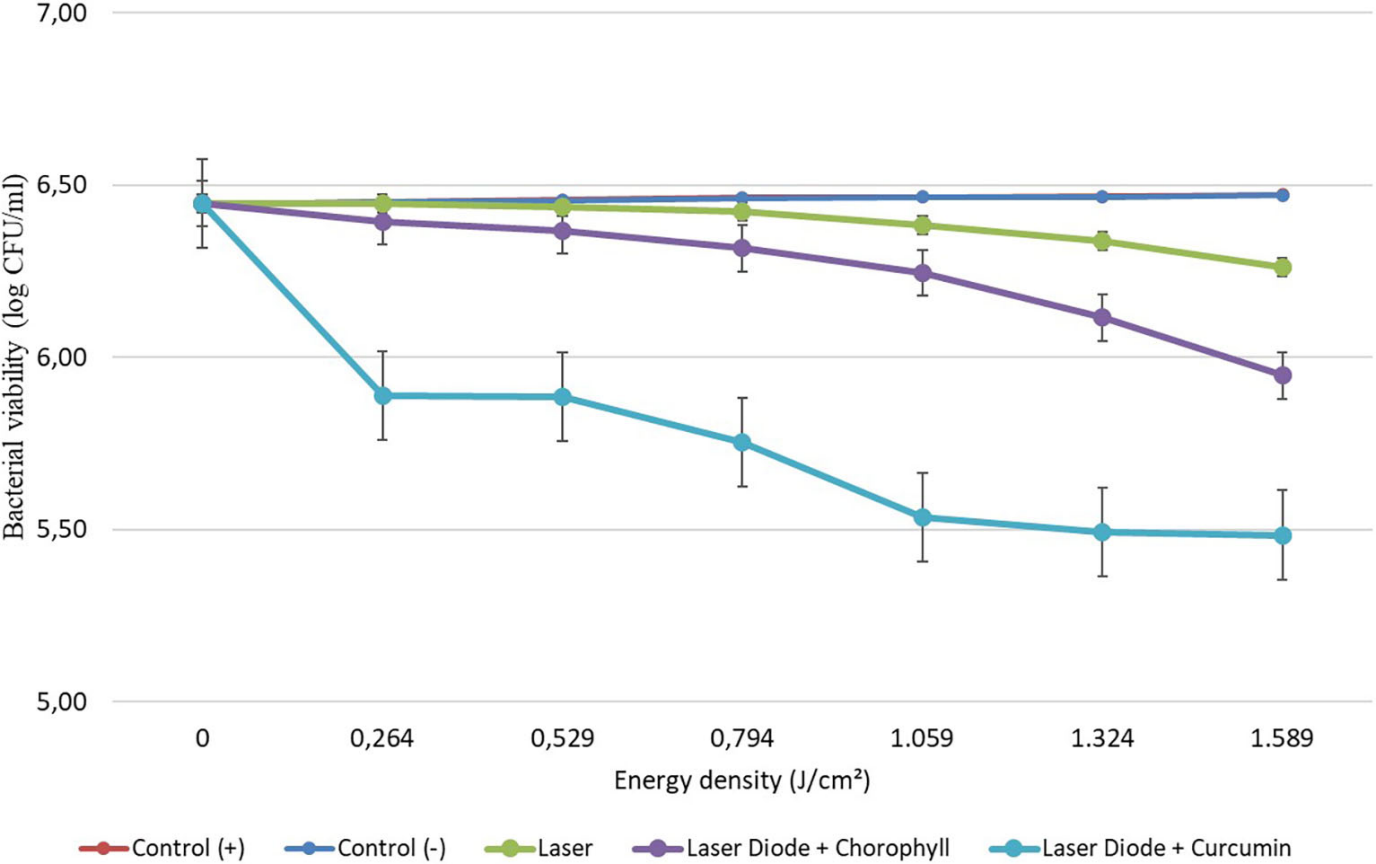
Graph of bacterial viability of *E. faecalis* in various treatments with and without the addition of curcumin and chlorophyll Medicago sativa L.

After that, antibacterial tests were carried out on *Aggregatibacter actinomycetemcomitans* and *Enterococcus faecalis* bacteria, which were exposed to a diode laser with and without a photosensitizer. So, the viability of the bacteria *Aggregatibacter actinomycetemcomitans* and *Enterococcus faecalis* is shown in Figures 2 and 3. Based on bacterial viability, the percentages of death of *A. actinomycetemcomitans* and *E. faecalis* bacteria by diode laser irradiation with the addition of curcumin photosensitizer and Medicago sativa L chlorophyll treatment are shown in Tables 2 and 3.

**Table 2.** Results of statistical analysis of *Enterococcus faecalis* bacteria.

Group	Irradiation time (s)	Energy density (J/cm ^2)^	N	% Bacterial death		Anova	
				Mean	Std.	Sig.	Conclusion
Laser	30	0.264 ^(1)^	4	2.61	0.90	0.00	There is a difference in meaning
	60	0.529 ^(1.2)^	4	4.83	1.74		
	90	0.794 ^(2)^	4	7.99	2.06		
	120	1.059 ^(3.4)^	4	15.65	1.46		
	150	1.324 ^(5)^	4	23.39	1.40		
	180	1.589 ^(7)^	4	35.81	2.09		
Lasers and Chlorophyll	30	0.264 ^(3)^	4	13.89	0.63	0.00	
	60	0.529 ^(4)^	4	18.68	1.69		
	90	0.794 ^(6)^	4	27.95	1.22		
	120	1.059 ^(7)^	4	39.35	0.75		
	150	1.324 ^(8)^	4	54.69	0.83		
	180	1.589 ^(9)^	4	69.27	1.74		
Lasers and Curcumin	30	0.264 ^(10)^	4	73.20	0.17	0.00	
	60	0.529 ^(10)^	4	73.29	0.28		
	90	0.794 ^(11)^	4	80.31	0.33		
	120	1.059 ^(12)^	4	88.12	0.33		
	150	1.324 ^(13)^	4	89.25	0.28		
	180	1.589 ^(13)^	4	89.42	0.20		

**Table 3.** Results of statistical analysis of *Aggregatibacter actinomycetemcomitans* bacteria.

Group	Irradiation time (s)	Energy density (J/cm ^2)^	N	% Bacterial death		Anova	
				Mean	Std.	Sig.	Conclusion
Laser	30	0.264 ^(1)^	4	1.45	2.57	0.00	There is a difference in meaning
	60	0.529 ^(2)^	4	8.82	1.58		
	90	0.794 ^(2.3)^	4	12.99	0.91		
	120	1.059 ^(3.4)^	4	16.73	1.07		
	150	1.324 ^(5)^	4	26.48	2.83		
	180	1.589 ^(7)^	4	41.99	1.24		
Lasers and Chlorophyll	30	0.264 ^(2)^	4	8.39	1.33	0.00	
	60	0.529 ^(4)^	4	20.80	2.49		
	90	0.794 ^(6)^	4	34.12	2.00		
	120	1.059 ^(6.7)^	4	37.96	2.46		
	150	1.324 ^(8)^	4	51.46	3.14		
	180	1.589 ^(9)^	4	62.96	2.01		
Lasers and Curcumin	30	0.264 ^(10)^	4	85.00	0.18	0.00	
	60	0.529 ^(11)^	4	86.14	0.20		
	90	0.794 ^(12)^	4	87.89	0.35		
	120	1.059 ^(12)^	4	88.68	0.18		
	150	1.324 ^(13)^	4	89.74	0.18		
	180	1.589 ^(13)^	4	89.82	0.29		

Based on the results of statistical tests, it was shown that diode laser irradiation with an energy density of 1.59 J/cm ^2^ gave a percentage of *E. faecalis* bacteria death of 36.7% without adding a photosensitizer. Then, the percentage of death of *E. faecalis* bacteria was 69.30% with the addition of PS chlorophyll Medicago sativa L. and 89.42% with the addition of PS curcumin. Meanwhile, the results of statistical tests on bacteria *A. actinomycetemcomitans* with diode laser irradiation at an energy density of 1.59 J/cm ^2^ gave the percentage of bacterial death of 35.81% without the addition of PS. Then, the death of *A. actinomycetemcomitans* was 64.39% with the addition of PS chlorophyll and 89.82% with the addition of PS curcumin.

## Discussion

The results of this study indicate that photodynamic inactivation (PDI) using curcumin as a photosensitizer (PS) is the most effective strategy for significantly reducing the viability of both *E. faecalis* and *A. actinomycetemcomitans*. This research was conducted using the PDI technique using a blue diode laser as a light source, chlorophyll Medicago sativa L and curcumin as PS to reduce bacteria *E. faecalis* and *A. actinomycetemcomitans.* The wavelength of light is an important factor in the photoinactivation process. The diode laser used in this study has a wavelength of 405 nm and an output power of 2.49 mW. The results of the characterization of power against time and temperature show the stability of power and temperature so that the temperature factor does not cause the death of bacteria.

PS is a light-sensitive molecule. Exogenous PS is PS that is added to assist the photoinactivation process. This study used exogenous PS chlorophyll Medicago sativa L and curcumin. Medicago sativa L chlorophyll absorbance used for a laser wavelength of 405 nm was 85.1% and for curcumin it was 80.64%. ^25^ The photoinactivation process occurs due to a photophysical mechanism initiated by the absorption of light by PS. The energy of the absorbed photon will cause the excitation of the electron to increase to a higher energy level. If the energy excitation state overlaps with the triplet excitation state, an intersystem crossing occurs, a spin reversal that places the electron in a triplet excited state and triggers a photochemical reaction.

**Figure 4.**
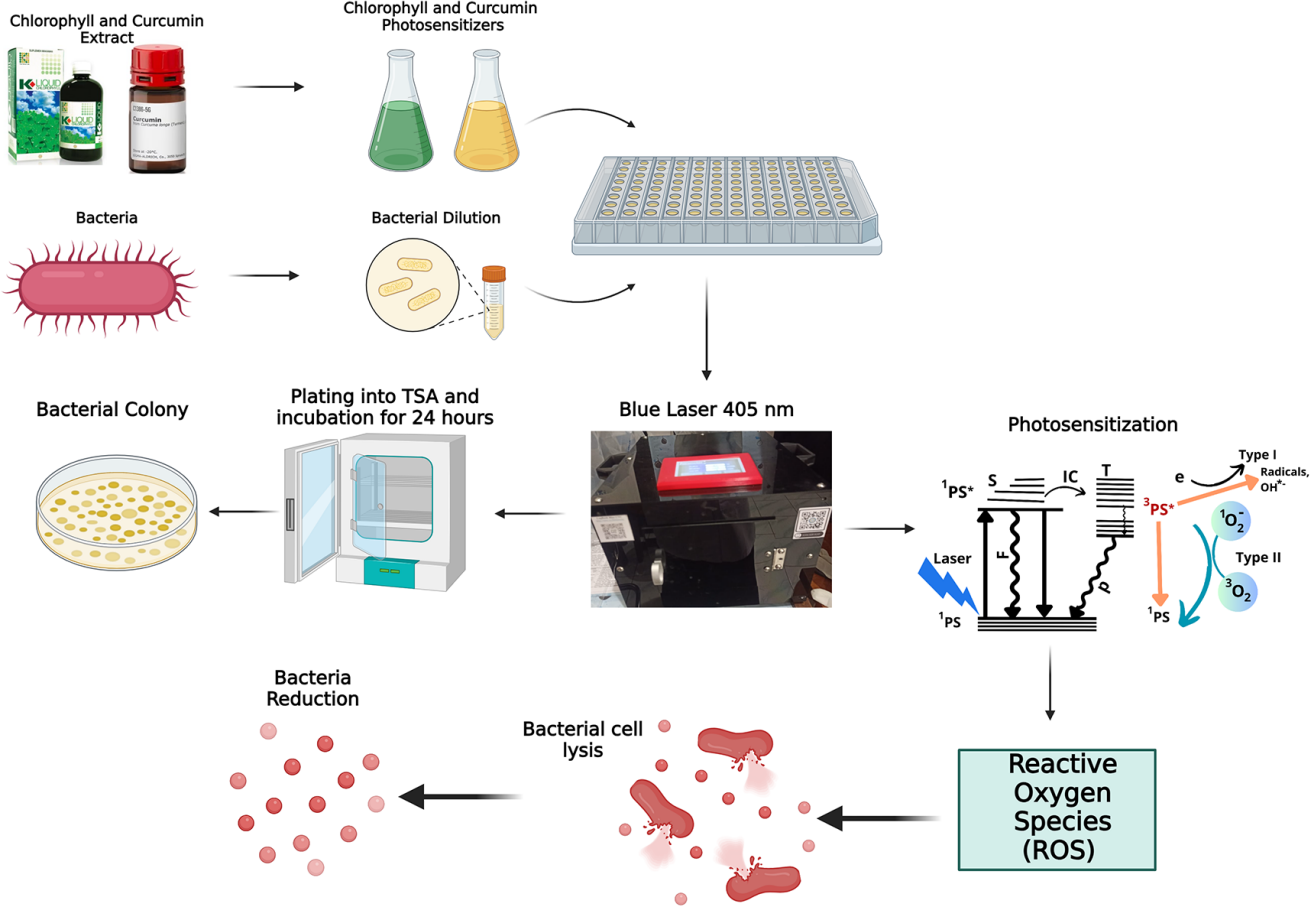
Mechanism of bacterial inactivation.

The mechanism of photodynamic inactivation (PDI) is illustrated in Figure 4. During photodynamic therapy, chlorophyll and curcumin act as photosensitizers. A photosensitizer is a solution that is able to absorb laser light optimally through a photophysical process. The first process that occurs is that the photosensitizer absorbs photon energy from a 405 nm laser diode, causing excitation from a low state ( ^1^PS) to an excited state ( ^1^PS*). In the ^1^PS* state, the photosensitizer can return to the ^1^PS state by emitting fluorescence or internal conversion. In addition, the photosensitizer can also convert to a more stable T nearest excitation level before finally returning to the ^1^PS state by emitting energy in the form of phosphorescence. This energy conversion is what requires oxygen through the photochemical process. Photochemical reactions are divided into two types. The first type is the transfer of electrons to a biological substrate in the form of a redox reaction and produces singlet oxygen. The second type is the transfer of energy to the triplet electrons to produce singlet oxygen. Singlet oxygen is radical. Reactive oxygen species (ROS) can damage the bacterial cell membrane and cause lysis. This is the earliest stage in bacterial cell death. Photochemical activity generates ROS and triplet oxygen radicals that oxidize unsaturated fatty acids, producing hydroperoxides. These radicals interfere with the synthesis of saturated fatty acids, causing harmful hydroperoxides known as lipid peroxidation. This disruption of the bacterial cell wall leads to cell lysis. ^25^ The photochemical reactions in PDI are generally of the second type.

The study’s results on *E. faecalis* bacteria showed a significant difference between treatments. Diode laser irradiation with an energy density of 1.59 J/cm ^2^ gave the percentage of bacterial death of *E. faecalis* 36.7% without the addition of PS, 69.30% with the addition of PS chlorophyll Medicago sativa L and 89.42% with the addition of PS curcumin. Meanwhile, in *A. actinomycetemcomitans* bacteria with energy density diode laser irradiation of 1.59 J/cm ^2^, the percentage of bacterial death was 35.81% with the addition of PS, 64.39% with the addition of PS chlorophyll Medicago sativa L and 89.82% with the addition of PS curcumin.

The results showed that adding PS curcumin increased the effectiveness of reducing *E. faecalis* and *A. actinomycetemcomitans* bacteria. PDI with PS curcumin was effectively used to reduce bacteria because its absorption followed endogenous porphyrins. ^26^ The addition of energy density will increase the reduction effect without and with the addition of PS Medicago sativa L ^19^ and curcumin. ^21^ In addition, the results showed that the administration of Medicago chlorophyll PS was more effective in reducing *E. faecalis* bacteria than *A. actinomycetemcomitans.* This is because the wall structure of *E. faecalis* bacteria is thinner than that of *A. actinomycetemcomitans.*

*A. actinomycetemcomitans* is a Gram-negative bacterium measuring 0.4-0.5 μm × 1.0-1.5 μm. ^27^ *Enterococcus faecalis* bacteria are Gram-positive bacteria with a cell wall thickness of about 40 nm. ^28^ The cell wall structure of gram-negative bacteria consists of lipopolysaccharides. lipoproteins, lipopolysaccharides, and peptidoglycans. The cell wall of gram-negative bacteria is more complex, so it is more difficult to penetrate antibacterial compounds. The cell wall structure of gram-positive bacteria is relatively simpler so that antibacterial compounds easily enter the cell. ^27^

In this study, PS curcumin has a higher percentage of bacterial death than PS chlorophyll (Medicago sativa L). This is in accordance with research conducted by Avianti *et al.* (2020) that PS curcumin in photodynamic therapy has a greater bacterial death effect than PS chlorophyll on *E. faecalis.* In addition, the research by Avianti *et al.* (2020) was also conducted at 60 and 90 seconds of irradiation duration. Then, it was proven that the duration of 90 seconds on all PS proved effective in increasing the death of *E. faecalis* bacteria. This is related to the nature of curcumin and its molecular structure, which enhances its function in photodynamic therapy. It shows that the longer the duration of laser irradiation given, the greater the bacterial death caused. ^23^

Research conducted by Balhaddad *et al.* (2020) showed that irradiation of S. mutans biofilm through 100 μg/mL TBO and an energy dose of ≈180 J/cm ^2^ resulted in a higher number of dead S. mutans colonies compared to the control (p < 0.001). Light energy dose and PS concentration optimize bacterial death. ^29^ Later, Pordel *et al.* (2023) reported that chlorhexidine and curcumin with a blue diode laser at 500 mW output power had the highest reduction in the number of S. mutans colonies (p < 0.001). The curcumin group was more effective than the riboflavin group. The results of the study by Pordel *et al*. (2023) showed that aPDT using curcumin as a photosensitizer plus a blue diode laser with an output power of 500 mW and a power density of 1.0 W/cm ^2^ at a wavelength of 445 nm can effectively reduce the colonies of S. mutans. ^30^ In addition, Ashtiani *et al*. (2024) also reported the results of their research that toluidine blue O (TBO) and phycocyanin (PC) activated with a 635 nm diode laser with a power density of 1592 W/cm ^2^ were more efficient in increasing the death of *E. faecalis* bacteria compared to a 636 W/cm ^2^ diode laser (p = 0.00). Light power density optimizes the reduction of *E. faecalis* bacteria. ^31^

In vivo studies are found in other journals. ^32^^,^^33^ The effect of bacterial photoinactivation on wounds in vivo due to photodynamic therapy in the red LED exposure group was 88%, the blue LED exposure group was 94%, and the red and blue LED combination exposure group was 95%. ^33^ Blue and red lasers combined with ozone treatment effectively accelerated wound healing of incisions infected with MRSA bacteria. ^34^ Thus, LED antimicrobial photodynamic therapy is effective for bacterial inactivation and accelerates wound healing in mice. Biofilm research is also found in other journals. ^33^^,^^34^ The difference in the reduction rate of PDT increased when ozone was added, thus helping biofilm reduction compared to PDT. ^33^ The results showed that the Chlo+Ozon+Laser combination treatment at 20 seconds of ozone exposure with 4 minutes of irradiation time caused a decrease in biofilm activity by 80.26%, which was the highest efficacy of all treatment groups. The combination of laser, chlorophyll, and lower ozone concentration increased the effectiveness of photodynamic inactivation. ^35^

## Conclusion

Based on statistical tests, the results of research on *E. faecalis* bacteria showed that laser irradiation with an energy density of 1.59 J/cm ^2^ gave a percentage of *E. faecalis* bacteria mortality of 36.7% without the addition of PS, 69.30% with the addition of PS chlorophyll Medicago sativa L and 89.42% with the addition of PS curcumin. Meanwhile, *A. actinomycetemcomitans* showed that the energy density diode laser irradiation of 1.59 J/cm ^2^ gave the percentage of bacterial death 35.81% without the addition of PS, 64.39% with the addition of PS chlorophyll Medicago sativa L and 89.82% with the addition of PS curcumin. So it can be concluded that the role of PS is significant for the success of PDI. The addition of PS curcumin increased the effectiveness of reducing bacteria *E. faecalis* and *A. actinomycetemcomitans* compared to chlorophyll Medicago sativa L.

## Author contributions

DA contributes to the data curation, methodology, validation, original draft preparation of the work and editing of the work. SDA contributes to the conception, methodology, analysis, funding acquisitions, project administration, supervision, validation, review, original draft preparation of the work and editing of the work. SRM, SBL, DZIN, and DAH contribute to the conception, data curation, methodology, investigation, validation, original draft preparation of the work and editing of the work. S contributes to the conception, methodology, investigation, analysis, supervision, validation, review, original draft preparation of the work and editing of the work. YS contributes to the conception, data curation, methodology, investigation, validation, original draft preparation of the work and editing of the work. AS contributes to the conception, methodology, analysis, supervision, validation, review, original draft preparation of the work and editing of the work.

## Data Availability

Open Science Framework: Antimicrobial Reduction Effect of Photodynamic Inactivation with The Addition Photosensitizer. https://doi.org/10.17605/OSF.IO/BEZ2R. ^36^ This project contains the following files:
•
Table 1. Laser energy density at various time exposure.docx (The laser parameters used in this research) •
Table 2. Results of statistical analysis of Enterococcus faecalis bacteria.docx •
Table 3. Results of statistical analysis of Aggregatibacter actinomycetemcomitans bacteria.docx • Bacterial Treatment Group Aggregatibacter actinomycetemcomitant
○ CFU Calculation Aggregatibacter actinomycetemcomitant.xlsx ○ Excel of Bacterial Viability.xls ○ Graph of Bacterial Viability.jpg
• Bacterial Treatment Group Enterococcus faecalis
○ CFU Calculation Bacterial Treatment Group Enterococcus faecalis.xlsx ○ Excel of Bacterial Viability.xls ○ Graph of Bacterial Viabilty.jpg Open Science Framework: Antimicrobial Reduction Effect of Photodynamic Inactivation with The Addition Photosensitizer. https://doi.org/10.17605/OSF.IO/BEZ2R. ^36^ This project contains the following extended data:
•
Figure 1. Graph of curcumin and chlorophyll absorption spectrum.jpg •
Figure 2. Graph of bacterial viability of A. actinomycetemcomitans in various treatments with and without the addition of curcumin and chlorophyll Medicago sativ.jpg •
Figure 3. Graph of bacterial viability of E. faecalis in various treatments with and without photosensitizer.jpg •
Figure 4. Mechanism of Bacterial Inactivation.png • Equipment used during the research.docx • The materials used during the research.docx • Treatment process.docx Data are available under the terms of the Creative Commons Attribution 4.0 International license (CC-BY 4.0).
