# Supplementary material for: Comparison of the antimicrobial reduction effect of photodynamic inactivation with the addition of chlorophyll and curcumin photosensitizer in Aggregatibacter actinomycetemcomitans and Enterococcus faecalis
Source: F1000Res. 2025 Nov 14;12:142. Originally published 2023 Feb 7. [Version 5] doi: 10.12688/f1000research.128483.5 (PMC12631066; doi:10.12688/f1000research.128483.5)
Supplement: Peer review history

# Reviewer report: Reviewer response for version 5

**DOI:** 10.5256/f1000research.189529.r432692

**Published:** 2025-11-19

**Authors:** Gaetano Isola

**Affiliations:** 1 University of Catania, Catania, Italy

The authors have well addressed all issues raised by the reviewer.

Is the work clearly and accurately presented and does it cite the current literature?

Partly

If applicable, is the statistical analysis and its interpretation appropriate?

Yes

Are all the source data underlying the results available to ensure full reproducibility?

Partly

Is the study design appropriate and is the work technically sound?

Partly

Are the conclusions drawn adequately supported by the results?

Yes

Are sufficient details of methods and analysis provided to allow replication by others?

Yes

Reviewer Expertise:

periodontology

I confirm that I have read this submission and believe that I have an appropriate level of expertise to confirm that it is of an acceptable scientific standard.

# Reviewer report: Reviewer response for version 4

**DOI:** 10.5256/f1000research.179507.r382890

**Published:** 2025-05-28

**Authors:** Gaetano Isola

**Affiliations:** 1 University of Catania, Catania, Italy

In the manuscript entitled: “Comparison of the antimicrobial reduction effect of photodynamic inactivation with the addition of chlorophyll and curcumin photosensitizer in Aggregatibacter actinomycetemcomitans and Enterococcus faecalis" the authors aimed to assess  PDI method with a 405 nm diode laser at various energy density with the addition PS curcumin or chlorophyll Alfalfa, as much as 1.6 mg/ml on A. actinomycetemcomitans and E. faecalis bacteria.

 The authors found that the energy density diode laser irradiation of 1.59 J/cm² gave the percentage of E. faecalis bacteria death 36.7% without PS, 69.30% with the addition of chlorophyll Medicago sativa L and 89.42% with the addition of curcumin. Meanwhile, the bacteria A. actinomycetemcomitans showed that the energy density diode laser irradiation of 1.59 J/cm² gave the percentage of bacterial death 35.81% without PS, 64.39% with the addition of chlorophyll Medicago sativa L and 89.82% with the addition of curcumin. PS was critical to the success of the PDI.

 The authors concluded that the addition of PS curcumin increased the effectiveness of reducing bacteria E. faecalis and A. actinomycetemcomitans compared to chlorophyll Medicago sativa L.

 Major comments:

 In general, the idea and innovation of this study regards the analysis of the link between mediator and periodontitis is interesting and novel because the role these aspects in medicine are validated but further studies on this topic could be an innovative issue in this field could be open a creative matter of debate in literature by adding new information. Moreover, there are few reports in the literature that studied this interesting topic with this kind of study design.

 The study was well conducted by the authors; However, there are some concerns to revise that are described below.

 The introduction section resumes the existing knowledge regarding the important factor linked with the relationship between periodontitis and systemic diseases.

 However, as the importance of the topic, the reviewer strongly recommends, before a further re-evaluation of the manuscript, to update the literature through read, discuss and must cites in the references with great attention all of those recent interesting articles, that helps the authors to better introduce and discuss the relationship and mediators of periodontitis peri-implant diseases and systemic diseases and how periodontal treatment could improve microbiome outcomes: 1) Isola G, et al.: 2025 (Ref 1) 2) Isola G, Polizzi A, Santagati M, Alibrandi A, et al 2025 (Ref 2)

 The authors should be better specified, at the end of the introduction section, the rationale of the study and the aim of the study. In the central section, should better clarify inclusions and exclusions criteria of the selected sample.

 Please better state the results obtained in the abstract.

 The discussion section appears well organized with the relevant paper that support the conclusions, even if the authors should better discuss the relationship regarding the by periodontitis in and risk of oxidative stress evolution in periodontitis patients. The conclusion should reinforce in light of the discussions.

 In conclusion, I am sure that the authors are fine clinicians who achieve very nice results with their adopted protocol. However, this study, in my view does not in its current form satisfy a very high scientific requirement for indexing in this journal and requests a revision before a further re-evaluation of the manuscript.

 Minor Comments:

 Abstract:

- Better formulate the abstract section by better describing the aim of the study

Introduction:

- Please refer to major comments

Discussion

- Please add a specific sentence that clarifies the results obtained in the first part of the discussion

Is the work clearly and accurately presented and does it cite the current literature?

Partly

If applicable, is the statistical analysis and its interpretation appropriate?

Yes

Are all the source data underlying the results available to ensure full reproducibility?

Partly

Is the study design appropriate and is the work technically sound?

Partly

Are the conclusions drawn adequately supported by the results?

Yes

Are sufficient details of methods and analysis provided to allow replication by others?

Yes

Reviewer Expertise:

periodontology

I confirm that I have read this submission and believe that I have an appropriate level of expertise to confirm that it is of an acceptable scientific standard, however I have significant reservations, as outlined above.

## References

[1] : Relationship between periodontitis and systemic diseases: A bibliometric and visual study. *Periodontol 2000* .2025; 10.1111/prd.12621 10.1111/prd.12621 39775963 PMC12842847

[2] : Effect of Nonsurgical Mechanical Debridement With or Without Chlorhexidine Formulations in the Treatment of Peri-Implant Mucositis. A Randomized Placebo-Controlled Clinical Trial. *Clin Oral Implants Res* .2025;36(5) : 10.1111/clr.14405 566-577 10.1111/clr.14405 39865359 PMC12066909

# Response

**Published:** 2025-10-09

**Authors:** Suryani Dyah Astuti

**Affiliations:** Airlangga University, Indonesia

**Reviewer Comment for Author:**

**Major comments:**

 In general, the idea and innovation of this study regard the analysis of the link between mediator and periodontitis is interesting and novel because the role these aspects in medicine are validated but further studies on this topic could be an innovative issue in this field could be open a creative matter of debate in literature by adding new information. Moreover, there are few reports in the literature that studied this interesting topic with this kind of study design.

 The study was well conducted by the authors; However, there are some concerns to revise that are described below.

 The introduction section resumes the existing knowledge regarding the important factor linked with the relationship between periodontitis and systemic diseases.

 However, as the importance of the topic, the reviewer strongly recommends, before a further re-evaluation of the manuscript, to update the literature through read, discuss and must cites in the references with great attention all of those recent interesting articles, that helps the authors to better introduce and discuss the relationship and mediators of periodontitis peri-implant diseases and systemic diseases and how periodontal treatment could improve microbiome outcomes: 1) Isola G, et al.: 2025 (Ref 1) 2) Isola G, Polizzi A, Santagati M, Alibrandi A, et al 2025 (Ref 2)

 The authors should be better specified, at the end of the introduction section, the rationale of the study and the aim of the study. In the central section, should better clarify inclusions and exclusions criteria of the selected sample.

 Please better state the results obtained in the abstract.

 The discussion section appears well organized with the relevant paper that support the conclusions, even if the authors should better discuss the relationship regarding the by periodontitis in and risk of oxidative stress evolution in periodontitis patients. The conclusion should reinforce in light of the discussions.

 In conclusion, I am sure that the authors are fine clinicians who achieve very nice results with their adopted protocol. However, this study, in my view does not in its current form satisfy a very high scientific requirement for indexing in this journal and requests a revision before a further re-evaluation of the manuscript.

**Response to reviewer:**

 We would like to say thank you for your valuable suggestion and the opportunity to revise the manuscript.  We already revised it.

**Minor Comments:**

**Abstract:**

 Better formulate the abstract section by better describing the aim of the study

**Response to reviewer:**

 Background Antibiotic resistance in oral pathogens such as *Aggregatibacter actinomycetemcomitans* and *Enterococcus faecalis* necessitates alternative antimicrobial strategies. Photodynamic inactivation (PDI) is a promising approach that utilizes a photosensitizer (PS), light, and oxygen to generate cytotoxic reactive oxygen species. This study evaluated the efficacy of two natural photosensitizers, curcumin and chlorophyll (from Medicago sativa L.), for the photodynamic inactivation of these bacteria. Methods Planktonic cultures of *A. actinomycetemcomitans* and *E. faecalis* were subjected to PDI using a 405 nm diode laser at various energy densities. The bacteria were treated with either curcumin (1.6 mg/mL) or chlorophyll (1.6 mg/mL) prior to laser irradiation. Bacterial viability was assessed using the colony-forming unit (CFU) assay. Groups without PS and without light served as controls. Results At an energy density of 1.59 J/cm², laser irradiation resulted in a reduction of 35.8% and 36.7% for *A. actinomycetemcomitans* and *E. faecalis*, respectively. The addition of chlorophyll significantly enhanced bacterial reduction to 64.4% and 69.3%. Curcumin demonstrated superior efficacy, achieving bacterial reduction of 89.8% and 89.4% for *A. actinomycetemcomitans* and *E. faecalis*, respectively. The differences between the PS groups and the control groups were statistically significant (p < 0.05). Conclusions Curcumin is a significantly more effective natural photosensitizer than chlorophyll from Medicago sativa L. for the photodynamic inactivation of *A. actinomycetemcomitans* and *E. faecalis*. These findings point to the prospect of curcumin-based PDI as a potent therapeutic alternative for combating resistant oral infections.

**Introduction:**

 Please refer to major comments

**Response to reviewer:**

 Periodontitis, a chronic inflammatory disease triggered by dysbiotic oral microbiome, has implications that extend far beyond the oral cavity. A recent bibliometric analysis highlights the deep and growing research interest in the strong relationship between periodontitis and systemic diseases, such as diabetes, cardiovascular disease, and adverse pregnancy outcomes. This well-established systemic relationship confirms that effective periodontitis management is not merely a dental concern but a critical component of overall public health. Consequently, there is an urgent need to develop innovative and effective therapeutic strategies to control periodontal biofilm and reduce the associated local and systemic inflammatory burden. Conventional treatments for inflammatory conditions, including peri-implant mucositis, often consist of non-surgical mechanical debridement enhanced with antiseptics like chlorhexidine. The limitations of these conventional treatments emphasize the necessity for exploring alternate therapy methods, including antimicrobial photodynamic inactivation.

**Discussion:**

 Please add a specific sentence that clarifies the results obtained in the first part of the discussion

**Response to reviewer:**

 The results of this study indicate that photodynamic inactivation (PDI) using curcumin as a photosensitizer (PS) is the most effective strategy for significantly reducing the viability of both *E. faecalis *and *A. actinomycetemcomitans*.

# Reviewer report: Reviewer response for version 3

**DOI:** 10.5256/f1000research.171310.r330190

**Published:** 2024-12-14

**Authors:** Shima Afrasiabi

**Affiliations:** 1 Tehran University of Medical Sciences, Tehran, Iran

-The culture conditions of A. actinomycetemcomitans differ from those of E. faecalis. The authors have not considered this case.

 - There is a duplicate data. Graph of the percentage of death of bacteria should be removed. (Fig 4, 5)

Is the work clearly and accurately presented and does it cite the current literature?

No

If applicable, is the statistical analysis and its interpretation appropriate?

Partly

Are all the source data underlying the results available to ensure full reproducibility?

Partly

Is the study design appropriate and is the work technically sound?

Partly

Are the conclusions drawn adequately supported by the results?

Yes

Are sufficient details of methods and analysis provided to allow replication by others?

No

Reviewer Expertise:

NA

I confirm that I have read this submission and believe that I have an appropriate level of expertise to confirm that it is of an acceptable scientific standard, however I have significant reservations, as outlined above.

# Response

**Published:** 2025-03-21

**Authors:** Suryani Dyah Astuti

**Affiliations:** Airlangga University, Indonesia

1. The culture conditions of A. actinomycetemcomitans differ from those of E. faecalis. The authors have not considered this case.

**Response to reviewer :**

 Thank you for your suggestion. This research only aims to determine the effect of irradiation or PDI treatment. So this study did not consider the incubation time or culture conditions of A. actinomycetemcomitans and E. faecalis bacteria.

 2. There is a duplicate data. Graph of the percentage of death of bacteria should be removed. (Fig 4, 5)

**Response to reviewer :**

 Thank you for your suggestion. We have changed it as you suggested.

# Reviewer report: Reviewer response for version 2

**DOI:** 10.5256/f1000research.163153.r308148

**Published:** 2024-08-21

**Authors:** Chenyu Jiang

**Affiliations:** 1 School of Optical and Electronic Information, Suzhou City University, Suzhou, China

This paper studied the photodynamic inactivation of chlorophyll and curcumin against two oral cavity related pathogens: *Aggregatibacter actinomycetemcomitans* and *Enterococcus faecalis* and found that *E. faecalis* bacteria exhibited 69.30% inactivation with the addition of chlorophyll Medicago sativa L and 89.42% inactivation with the addition of curcumin. Meanwhile, the bacteria *A. actinomycetemcomitans* presented 64.39% inactivation with the addition of chlorophyll Medicago sativa L and 89.82% inactivation with the addition of curcumin. PS was critical to the success of the PDI.

 Here are some comments:

1. The introduction did mention brief introduction of chlorophyll and curcumin but advantages of both compound as PS compared to other PS are encouraged to include in the introduction.
2. The figure 1 and figure 2 can be combined into one figure and distinguish two sample with different color or shape considering both figures are the absorption property.
3. The author did mention the there is a dilution factor but also mentioned that the treated sample was placed into incubator overnight directly. Please clarify this part.
4. The generation of ROS is encouraged to confirm via whatever chemical method, EPR, or photoluminescence. This study did mention that there are two type of ROS but none of them are confirmed under chlorophyll and curcumin illuminated by diode laser.

Is the work clearly and accurately presented and does it cite the current literature?

Yes

If applicable, is the statistical analysis and its interpretation appropriate?

Partly

Are all the source data underlying the results available to ensure full reproducibility?

Partly

Is the study design appropriate and is the work technically sound?

Yes

Are the conclusions drawn adequately supported by the results?

Yes

Are sufficient details of methods and analysis provided to allow replication by others?

Partly

Reviewer Expertise:

Photodynamic inactivation

I confirm that I have read this submission and believe that I have an appropriate level of expertise to confirm that it is of an acceptable scientific standard, however I have significant reservations, as outlined above.

# Response

**Published:** 2024-09-01

**Authors:** Suryani Dyah Astuti

**Affiliations:** Airlangga University, Indonesia

Reviewer 3:

 Reviewer Comment For Author:

 1. The introduction did mention brief introduction of chlorophyll and curcumin but advantages of both compound as PS compared to other PS are encouraged to include in the introduction.

**Response to reviewer :**

 Chlorophyll acts as a photosensitizer because it is naturally able to absorb light [17]. The chemical structure of chlorophyll consists of a porphyrin ring that serves as the core framework, and long hydrophobic side chains. The bioactivity of chlorophyll is due to its ability to act as an antioxidant, antimutagen, and anticarcinogen. Its unique chemical structure allows chlorophyll to capture harmful free radicals, reduce DNA damage, and modulate cellular processes involved in disease progression. In addition, its hydrophobic side chains facilitate interactions with biological membranes, affecting cellular uptake and signaling pathways [18].

 Curcumin is a curcuminoid compound with yellow pigment in turmeric rhizome, which is antitumor, antioxidant, anticarcinogenic, anti-inflammatory, antiviral, antifungal, antispasmodic, and hepatoprotective [19]. The absorption spectrum of curcumin is in the wavelength range of 375-475 nm [20]. The advantage of curcumin as PS is that there are reactive functional groups, including ketone and phenol groups, that play a role in phototherapeutic activity. Phenol groups play an important role in the interaction of curcumin and biological membranes of bacteria, because the attachment of hydrogen groups plays an important role [21].

 2. The figure 1 and figure 2 can be combined into one figure and distinguish two sample with different color or shape considering both figures are the absorption property.

**Response to reviewer :**

 We have transformed Images 1 and 2 into one image.

***Figure 1. Graph of curcumin and chlorophyll absorption spectrum***

 3. The author did mention the there is a dilution factor but also mentioned that the treated sample was placed into incubator overnight directly. Please clarify this part.

**Response to reviewer :**

 Bacteria *A. actinomycetemcomitans* ATCC 43718 and *E. faecalis *ATCC 29212 were cultured in tryptone soy broth (TSB). Then, it was incubated for 24 hours at 37 ^o^C until the colonies reached ~108 CFU/mL or 1.0 McFarland standard.

 4. The generation of ROS is encouraged to confirm via whatever chemical method, EPR, or photoluminescence. This study did mention that there are two type of ROS but none of them are confirmed under chlorophyll and curcumin illuminated by diode laser

**Response to reviewer :**

 The mechanism of photodynamic inactivation (PDI) is illustrated in Figure 6. During photodynamic therapy, chlorophyll and curcumin act as photosensitizers. A photosensitizers is a solution that is able to absorb laser light optimally through a photophysical process. The first process that occurs is that the photosensitizer absorbs photon energy from a 405 nm laser diode, causing excitation from a low state ( ^1^PS) to an excited state ( ^1^PS*). In the ^1^PS* state, the photosensitizer can return to the ^1^PS state by emitting fluorescence or internal conversion. In addition, the photosensitizer can also convert to a more stable T nearest excitation level before finally returning to the ^1^PS state by emitting energy in the form of phosphorescence. This energy conversion is what requires oxygen through the photochemical process. Photochemical reactions are divided into two types. The first type is the transfer of electrons to a biological substrate in the form of a redox reaction and produces singlet oxygen. The second type is the transfer of energy to the triplet electrons to produce singlet oxygen. Singlet oxygen is radical. Reactive oxygen species (ROS) can damage the bacterial cell membrane and cause lysis. This is the earliest stage in bacterial cell death. Photochemical activity generates ROS and triplet oxygen radicals that oxidize unsaturated fatty acids, producing hydroperoxides. These radicals interfere with the synthesis of saturated fatty acids, causing harmful hydroperoxides known as lipid peroxidation. This disruption of the bacterial cell wall leads to cell lysis [18]. The photochemical reactions in PDI are generally of the second type.

# Reviewer report: Reviewer response for version 2

**DOI:** 10.5256/f1000research.163153.r308151

**Published:** 2024-08-06

**Authors:** Shima Afrasiabi

**Affiliations:** 1 Tehran University of Medical Sciences, Tehran, Iran

I have the following comments:

 -The culture conditions of *A. actinomycetemcomitans* differ from those of *E. faecalis*. The authors have not considered this case.

 -In the discussion section, studies that share common characteristics such as similar PS or similar bacteria should be used. In addition, the use of different energy doses is not discussed. Please search again and refer to the articles below;

 (i) Balhaddad AA et al. (2020 [Ref-1]) [doi.org/10.3390/ijms21207612]

 (ii) Pordel E et al. (2023 [Ref - 2]) [doi.org/10.3390/biomedicines11082248]

 (iii) Ashtiani AS et al. (2024 [Ref - 3])  [doi.org/10.1016/j.pdpdt.2024.104053]

 - Figures 3, 4, 5, and 6 should be summarized in a Table by mentioning SD.

**Discussion**

 Line 3; “The laser diode used in this study” should be diode laser.

 Photodynamic Inactivation, please use lowercase letters.

 - The manuscript contains many writing problems.

Is the work clearly and accurately presented and does it cite the current literature?

No

If applicable, is the statistical analysis and its interpretation appropriate?

Partly

Are all the source data underlying the results available to ensure full reproducibility?

Partly

Is the study design appropriate and is the work technically sound?

Partly

Are the conclusions drawn adequately supported by the results?

Yes

Are sufficient details of methods and analysis provided to allow replication by others?

No

Reviewer Expertise:

Photodynamic therapy

I confirm that I have read this submission and believe that I have an appropriate level of expertise to confirm that it is of an acceptable scientific standard, however I have significant reservations, as outlined above.

## References

[1] : Light Energy Dose and Photosensitizer Concentration Are Determinants of Effective Photo-Killing against Caries-Related Biofilms. *Int J Mol Sci* .2020;21(20) : 10.3390/ijms21207612 10.3390/ijms21207612 33076241 PMC7589159

[2] : The Effect of Different Output Powers of Blue Diode Laser along with Curcumin and Riboflavin against Streptococcus mutans around Orthodontic Brackets: An In Vitro Study. *Biomedicines* .2023;11(8) : 10.3390/biomedicines11082248 10.3390/biomedicines11082248 37626744 PMC10452080

[3] : In vitro antibiofilm effect of different irradiation doses in infected root canal model. *Photodiagnosis Photodyn Ther* .2024;46: 10.1016/j.pdpdt.2024.104053 104053 10.1016/j.pdpdt.2024.104053 38499277

# Response

**Published:** 2024-09-01

**Authors:** Suryani Dyah Astuti

**Affiliations:** Airlangga University, Indonesia

**Reviewer 2:**

**Reviewer Comment For Author:**

 I have the following comments:

 -The culture conditions of A. actinomycetemcomitans differ from those of E. faecalis. The authors have not considered this case.

**Response to reviewer :**

 In addition, the results showed that the administration of Medicago chlorophyll PS was more effective in reducing E. faecalis bacteria than A. actinomycetemcomitans. This is because the wall structure of E. faecalis bacteria is thinner than A. actinomycetemcomitans.

 A. actinomycetemcomitans is a Gram-negative bacterium measuring 0.4-0.5 µm × 1.0-1.5 µm. [25]. Enterococcus faecalis bacteria are Gram-positive bacteria with a cell wall thickness of about 40 nm [26]. The cell wall structure of gram-negative bacteria consists of lipopolysaccharides. lipoproteins, lipopolysaccharides, and peptidoglycans. The cell wall of gram-negative bacteria is more complex, so it is more difficult to penetrate antibacterial compounds. The cell wall structure of gram-positive bacteria is relatively simpler so that antibacterial compounds easily enter the cell [25].

**Reviewer Comment For Author:**

 -In the discussion section, studies that share common characteristics such as similar PS or similar bacteria should be used. In addition, the use of different energy doses is not discussed. Please search again and refer to the articles below;

 (i) Balhaddad AA et al. (2020 [Ref-1]) [doi.org/10.3390/ijms21207612]

 (ii) Pordel E et al. (2023 [Ref - 2]) [doi.org/10.3390/biomedicines11082248]

 (iii) Ashtiani AS et al. (2024 [Ref - 3])  [doi.org/10.1016/j.pdpdt.2024.104053]

**Response to reviewer :**

 In this study, PS curcumin has a higher percentage of bacterial death than PS chlorophyll (Medicago sativa L). This is in accordance with research conducted by Avianti *et al.* (2020) that PS curcumin in photodynamic therapy has a greater bacterial death effect than PS chlorophyll on E. faecalis. In addition, the research by Avianti *et al.* (2020) was also conducted at 60 and 90 seconds of irradiation duration. Then, it was proven that the duration of 90 seconds on all PS proved effective in increasing the death of E. faecalis bacteria. This is related to the nature of curcumin and its molecular structure, which enhances its function in photodynamic therapy. It shows that the longer the duration of laser irradiation given, the greater the bacterial death caused [21].

 Research conducted by Balhaddad *et al.* (2020), showed that irradiation of S. mutans biofilm through 100 μg/mL TBO and an energy dose of ≈180 J/cm2 resulted in a higher number of dead S. mutans colonies compared to the control (p < 0.001). Light energy dose and PS concentration optimize bacterial death [27]. Later, Pordel *et al.* (2023) reported that chlorhexidine and curcumin with a blue diode laser at 500 mW output power had the highest reduction in the number of S. mutans colonies (p < 0.001). The curcumin group was more effective than the riboflavin group. The results of the study by Pordel *et al.* (2023) showed that aPDT using curcumin as a photosensitizer plus a blue diode laser with an output power of 500 mW and a power density of 1.0 W/cm2 at a wavelength of 445 nm can effectively reduce the colonies of S. mutans [28]. In addition, Ashtiani et al. (2024) also reported the results of their research that toluidine blue O (TBO) and phycocyanin (PC) activated with a 635 nm diode laser with a power density of 1592 W/cm2 were more efficient in increasing the death of E. faecalis bacteria compared to a 636 W/cm2 diode laser (p = 0.00). Light power density optimizes the reduction of E. faecalis bacteria [29].

**Reviewer Comment For Author:**

 - Figures 3, 4, 5, and 6 should be summarized in a Table by mentioning SD.

**Response to reviewer :**

 We have added 2 Tables to summarize the data from Figures 3,4,5, and 6.

**Reviewer Comment For Author:**

 Discussion

 Line 3; “The laser diode used in this study” should be diode laser.

**Response to reviewer :**

 The diode laser used in this study has a wavelength of 405 nm and an output power of 2.49 mW.

**Reviewer Comment For Author:**

 Photodynamic Inactivation, please use lowercase letters.

**Response to reviewer :**

 The mechanism of photodynamic inactivation (PDI) is illustrated in Figure 6.

**Reviewer Comment For Author:**

 - The manuscript contains many writing problems.

**Response to reviewer :**

 Thank you for your suggestion. We have changed it as you suggested.

# Reviewer report: Reviewer response for version 1

**DOI:** 10.5256/f1000research.141076.r221717

**Published:** 2024-02-13

**Authors:** Min Wu

**Affiliations:** 1 Wellman Center for Photomedicine, Massachusetts General Hospital, Department of Dermatology, Harvard Medical School, Harvard University, Boston, Massachusetts, USA

This paper is about comparison of the antimicrobial reduction effect of two PSs in different bacteria related to oral cavity causing various diseases such as periodontitis and endodontics.

             I think the purpose and design of this manuscript is quite clear but a little bit too simple.

             I think it would be better if in vivo study is included to make the work more solid. For in vitro study, I think the authors can also add different types of ROS probe and even perform the biofilm experiment to support their conclusions.

 This manuscript focused on two pathogens highly related to various oral diseases. They used photodynamic inactivation (PDI) to deal with these pathogens. The photosensitizer are chlorophyll and curcumin. The light source is aBL (405nm), and treatment is aPDI. The data only include CFU which is gold standard and survival rate. They compared between different groups including light alone and negative control. The conclusions are also very clear.

Is the work clearly and accurately presented and does it cite the current literature?

Yes

If applicable, is the statistical analysis and its interpretation appropriate?

Yes

Are all the source data underlying the results available to ensure full reproducibility?

Yes

Is the study design appropriate and is the work technically sound?

Partly

Are the conclusions drawn adequately supported by the results?

Partly

Are sufficient details of methods and analysis provided to allow replication by others?

Yes

Reviewer Expertise:

Light-based therapy for both bench work and clinical work

I confirm that I have read this submission and believe that I have an appropriate level of expertise to confirm that it is of an acceptable scientific standard, however I have significant reservations, as outlined above.

# Response

**Published:** 2024-02-26

**Authors:** Suryani Dyah Astuti

**Affiliations:** Airlangga University, Indonesia

Thanks for valuable comments and suggestions for our manuscript. We have revised the manuscript according to the reviewers’ comments and answered them accurately. Some parts of the manuscript have been changed.

 1. The purpose and design of this manuscript is quite clear but a little bit too simple.

 Provide further comments here:

 We would like to say thank you for your valuable suggestion and the opportunity to revise the manuscript.  We already revised it.

 The purpose of this manuscript:

 Continuing previous studies [15,16,18], this study aims to compare the effectiveness of antimicrobial reduction from photodynamic inactivation (PDI) on bacteria *A. actinomycetemcomitans *and *E. faecalis* with PS curcumin and chlorophyll Medicago sativa L. using a 405 nm diode laser. Diode laser irradiation was carried out at various lengths of irradiation time, namely (30, 60, 90, 120, 150, and 180) seconds. In addition, this study aims to determine the effective laser irradiation time to reduce the bacteria A. actinomycetemcomitans and E. faecalis with PS curcumin and chlorophyll Medicago sativa L.

 The design of this manuscript:

 CFU/ml was calculated for each treatment using Equation 2. Furthermore, the percentage of bacterial reduction was calculated using Equation 3 based on the control group. The results of bacterial reduction were statistically analyzed using the Two Way Anova Factorial test using IBM SPSS Statistics Version 21 to determine the effect of each factor and its interaction between factors. The test usually requires distributed and at least interval scale data, with significant differences determined as P<α=0.05. Tukey's post hoc test and Kolmogorov-Smirnov test were used to check the normality of the data.

 2. I think it would be better if in vivo study is included to make the work more solid. For in vitro study, I think the authors can also add different types of ROS probe and even perform the biofilm experiment to support their conclusions.

 Provide further comments here:

 We would like to say thank you for your valuable suggestion and the opportunity to revise the manuscript.  We already revised it.

 The mechanism of Photodynamic Inactivation (PDI) is illustrated in Figure 7. Photochemical reactions are divided into two types. The first type is the transfer of electrons to a biological substrate in the form of a redox reaction and produces singlet oxygen. The second type is the transfer of energy to the triplet electrons to produce singlet oxygen. Singlet oxygen is a radical. Reactive Oxygen Species (ROS) can damage the bacterial cell membrane and cause lysis. This is the earliest stage in bacterial cell death. Photochemical activity generates ROS and triplet oxygen radicals that oxidize unsaturated fatty acids, producing hydroperoxides. These radicals interfere with the synthesis of saturated fatty acids, causing harmful hydroperoxides known as lipid peroxidation. This disruption of the bacterial cell wall leads to cell lysis [18]. The photochemical reactions in PDI are generally of the second type.

 In vivo studies are found in other journals [20,21]. The effect of bacterial photoinactivation on wounds in vivo due to photodynamic therapy in the red LED exposure group was 88%, the blue LED exposure group was 94%, and the red and blue LED combination exposure group was 95% [20]. Blue and red lasers combined with ozone treatment effectively accelerated wound healing of incisions infected with MRSA bacteria [21]. Thus, LED antimicrobial photodynamic therapy is effective for bacterial inactivation and accelerates wound healing in mice. Biofilm research is also found in other journals [22,23]. The difference in the reduction rate of PDT increased when ozone was added, thus helping biofilm reduction compared to PDT [22]. The results showed that the Chlo+Ozon+Laser combination treatment at 20 seconds of ozone exposure with 4 minutes irradiation time caused a decrease in biofilm activity by 80.26%, which was the highest efficacy of all treatment groups. The combination of laser, chlorophyll, and lower ozone concentration increased the effectiveness of photodynamic inactivation [24].
